# Tumor-Activated Neutrophils Promote Lung Cancer Progression through the IL-8/PD-L1 Pathway

**DOI:** 10.2174/0115680096337237240909101904

**Published:** 2024-10-01

**Authors:** Yiping Zheng, Jianfeng Cai, Qiuhong Ji, Luanmei Liu, Kaijun Liao, Lie Dong, Jie Gao, Yinghui Huang

**Affiliations:** 1 Department of Respiratory and Critical Care Medicine, Nanping First Hospital Affiliated to Fujian Medical University, Nanping, Fujian, 353006, China;; 2 Department of Clinical Medicine, Nanping First Hospital Affiliated to Fujian Medical University, Nanping, Fujian, 353006, China;; 3 Department of Gastrointestinal Surgery, Nanping First Hospital Affiliated to Fujian Medical University, Nanping, Fujian, 353006, China

**Keywords:** Lung cancer, neutrophils, IL-8, PD-L1, immune escape, tumor microenvironment

## Abstract

**Background:**

Lung cancer remains a major global health threat due to its complex microenvironment, particularly the role of neutrophils, which are crucial for tumor development and immune evasion mechanisms. This study aimed to delve into the impact of lung cancer cell-conditioned media on neutrophil functions and their potential implications for lung cancer progression.

**Methods:**

Employing *in vitro* experimental models, this study has analyzed the effects of lung cancer cell-conditioned media on neutrophil IL-8 and IFN-γ secretion, apoptosis, PD-L1 expression, and T-cell proliferation by using techniques, such as ELISA, flow cytometry, immunofluorescence, and CFSE proliferation assay. The roles of IL-8/PD-L1 in regulating neutrophil functions were further explored using inhibitors for IL-8 and PD-L1.

**Results:**

Lung cancer cell lines were found to secrete higher levels of IL-8 compared to normal lung epithelial cells. The conditioned media from lung cancer cells significantly reduced apoptosis in neutrophils, increased PD-L1 expression, and suppressed T-cell proliferation and IFN-γ secretion. These effects were partially reversed in the presence of IL-8 inhibitors in Tumor Tissue Culture Supernatants (TTCS), while being further enhanced by IL-8. Both apoptosis and PD-L1 expression in neutrophils demonstrated dose-dependency to TTCS. Additionally, CFSE proliferation assay results further confirmed the inhibitory effect of lung cancer cell-conditioned media on T-cell proliferation.

**Conclusion:**

This study has revealed lung cancer cell-conditioned media to modulate neutrophil functions through regulating factors, such as IL-8, thereby affecting immune regulation and tumor progression in the lung cancer microenvironment.

## 1 INTRODUCTION

Lung cancer remains one of the major global health threats, with its high incidence and mortality rates posing a severe public health issue [1-5]. Despite significant advancements in diagnostic and therapeutic techniques in recent years, the overall survival rate for lung cancer patients remains dismal [6-9]. Surgical resection, radiotherapy, and chemotherapy, while being primary treatment modalities, offer limited efficacy for advanced-stage patients and are often accompanied by severe side effects [2, 10-12]. With a deeper understanding of the molecular mechanisms of lung cancer, targeted therapy and immunotherapy have emerged as new hopes for treatment, yet they face challenges of uncertain efficacy and resistance development [10, 13, 14]. Therefore, delving into the pathogenesis of lung cancer, exploring more effective treatment strategies, and enhancing early diagnosis are imperative needs in current lung cancer research and therapy.

Research into the lung cancer microenvironment has provided a new perspective for understanding the complex biological behavior of lung cancer, encompassing tumor cells, surrounding vasculature, immune cells, extracellular matrix, and various cytokines and chemical signals, all constituting a complex network that influences tumor development and treatment response [15-20]. Immune cells, such as T cells, B cells, macrophages, dendritic cells, natural killer cells, and neutrophils, play significant roles in the lung cancer microenvironment [21-24]. They maintain immune surveillance and defense mechanisms in healthy tissues, but are often manipulated and altered by tumor cells in the lung cancer microenvironment, thereby promoting tumor growth and spread [25].

In the lung cancer microenvironment, neutrophils exhibit a complex and multifaceted role, not only serving as the first line of immune defense, but also playing a crucial role in lung cancer development and immune evasion [26-28]. Neutrophils can be induced by tumor cells to enter the tumor microenvironment, transforming into Tumor-associated Neutrophils (TANs) with dual characteristics [29-31]. Recent studies confirm that they can directly kill tumor cells by releasing reactive oxygen species and proteases or limit tumor cell spread by forming Neutrophil Extracellular Traps (NETs) [32]. Conversely, TANs can also promote tumor growth and metastasis by fostering inflammation, supporting angiogenesis, and remodeling the extracellular matrix [33, 34]. Additionally, neutrophils can directly or indirectly inhibit T cell activity by expressing immune checkpoint molecules, like PD-L1, or secreting immunosuppressive factors, thereby aiding tumor cells in evading immune surveillance [35]. Thus, a deeper understanding of the multifaceted roles of neutrophils in the lung cancer microenvironment is crucial for revealing lung cancer development mechanisms and developing new therapeutic strategies.

Recent explorations into key molecules have identified a potential link between IL-8 and PD-L1 as critical in the lung cancer microenvironment [36-38]. Studies, such as those by Horn *et al*., indicate that IL-8 and PD-L1 collaboratively participate in regulating tumor immune evasion and cellular plasticity [39]. IL-8, a typical pro-inflammatory cytokine, not only plays a role in attracting and activating neutrophils, but also exacerbates lung cancer malignancy by promoting angiogenesis and tumor cell migration and proliferation [40]. The expression of PD-L1 is key to tumor immune evasion strategies, inhibiting T cell activation and proliferation by binding to PD-1 on T cell surfaces, thus aiding tumor cells in evading immune system attacks [41-44]. In the lung cancer microenvironment, IL-8 and PD-L1 not only act independently, but their expression and function may also influence each other, collectively shaping an immunosuppressive tumor microenvironment, thereby affecting lung cancer development and treatment response [45]. While the importance of both has been widely recognized, their specific mechanisms of action and interactions in the lung cancer microenvironment remain hot research topics [46]. For example, IL-8 might affect PD-L1 expression and function by influencing immune cells, like neutrophils, in the tumor microenvironment [47], and conversely, the expression and regulation of PD-L1 might also be affected by cytokines, like IL-8 [48]. However, there are still unknown aspects regarding the role and regulatory mechanisms of neutrophils in lung cancer, limiting the understanding of lung cancer's full development and hindering the development of more effective treatment strategies.

This study aimed to delve into the impact of lung cancer cell-conditioned media on neutrophil functions, attempting to reveal the role and regulatory mechanisms of neutrophils in the lung cancer microenvironment. By exploring the interplay between TTCS/PD-L1/T cells and discussing the mechanism of IL-8 within this context, we hope to provide new insights into the immune regulatory mechanisms of lung cancer, especially offering new ideas and strategies for personalized and precision medicine.

## 2 MATERIALS AND METHODS

### 2.1 Cell Culture and Drug Treatment

Normal lung epithelial cells, BEAS-2B, and lung cancer cell lines, A549, HCC827, H1299, and Jurkat, were obtained from the American Type Culture Collection (ATCC) and cultured in RPMI-1640 medium (MeilunBio, Dalian, China) supplemented with 10% Fetal Bovine Serum (FBS, Cell-Box Biological Products Trading Co., Ltd, Hong Kong, China) and 1% penicillin-streptomycin (MeilunBio, Dalian, China). The supernatant purified from the A549 cell culture medium referred to as Tumor Tissue Culture Supernatant (TTCS), the supernatant purified from BEAS-2B cell culture medium referred to as Non-tumor Tissue Culture Supernatant (NTCS), and the culture medium were collected after 48 hours of cell passage.

Neutrophils were isolated from healthy volunteers using Histopaque-1077 (Solarbio, Beijing, China) according to the manufacturer's instructions and cultured with 50% TTCS or NTCS for 16 hours, followed by washing three times with complete RPMI-1640, and neutrophils cultured in RPMI-1640 medium served as control (Neu). There were 22 healthy volunteers, all of whom underwent routine blood tests, and the test data were found to be normal; the collected Neu were mixed and used. Interventions with IL-8 (100ng/ml, Novoprotein, Suzhou, China), anti-IL-8 (10μg/ml, BS-0780R, Bioss, Beijing, China), phytohemagglutinin-L (PHA-L, 00-4977-93, eBioscience™, CA, USA), a T-cell mitogen that stimulates T-cell proliferation, or D-PPA1 (6515, Bio-Techne, R&D Systems, Minneapolis, USA), a PD-L1 inhibitor, were conducted for 16 hours. After the Neu cell count, PBS was added to the suspension, and the Neu cell concentration was 1×10^6^/ml. The study was approved by the Ethics Committee of Nanping First Hospital (approval number: NPSY2021120003).

### 2.2 Enzyme-linked Immunosorbent Assay (ELISA) Detection

Samples were collected from the supernatants of lung cancer cell lines and neutrophils were cultured and stored at -80°C until use. IL-8 (MM-1558H2) and IFN-γ (MM-0033H2) kits from Jiangsu Meimian Industrial Co., Ltd (Jiangsu, China) were used according to the manufacturer's instructions. The ELISA plates were incubated at room temperature for a specific time, washed multiple times to remove unbound antibodies or antigens, and then substrates were added for color development. The absorbance was measured using a Multiscan MK3 enzyme reader (Thermo Fisher Scientific), and the concentrations of IL-8 and IFN-γ in the samples were calculated based on the standard curve.

### 2.3 Flow Cytometry Detection

For apoptosis: After various treatments, neutrophils were collected and incubated with Annexin V-FITC (5 μl, Meilune) and PI (10 μl, Meilune) in the dark for 15 minutes at 22°C. After washing with phosphate-buffered Saline (PBS, Meilune), cells were analyzed using a FACSCalbur flow cytometer (BD Biosciences, San Jose, CA, USA).

For PD-L1 detection: Treated neutrophils were fixed on slides, permeabilized with 0.1% Triton X-100, and then incubated with a specific fluorescently labeled antibody against PD-L1 in the dark for 1 hour. Afterward, cells were stained with a DAPI-containing anti-fade reagent (Meilune) for nuclear visualization. Finally, cells were observed under a fluorescence inverted microscope (Ts2-FL, Nikon, Japan), and the fluorescence intensity of different treatment groups was qualitatively analyzed for PD-L1 expression changes.

### 2.4 Immunofluorescence (IF) Detection

Treated neutrophils were fixed on slides with 4% paraformaldehyde (Aladdin, Shanghai, China) for 15 minutes at room temperature. After washing with PBS, cells were permeabilized with 0.1% Triton X-100 for 10 minutes. Subsequently, cells were incubated in 5% Bovine Serum Albumin (BSA) and PBS for 30 minutes, followed by overnight incubation at 4°C with FITC-labeled PD-L1 antibody (374510, BioLegend, CA, USA). Afterward, cells were stained with DAPI-containing anti-fade reagent (Meilune) for nuclear visualization and observed under a fluorescence-inverted microscope. Fluorescence intensity was quantitatively analyzed using ImageJ software (National Institutes of Health (NIH), Bethesda, MD, USA).

### 2.5 Carboxyfluorescein diacetate Succinimidyl Ester (CFSE) proliferation detection

The CFSE Cell Division Tracker Kit was used for proliferation detection according to the manufacturer's instructions. Briefly, cells were mixed with CFSE and incubated at 37°C for 10 minutes. The reaction was then quenched with 5 times the original staining volume of 1640 containing 10% FBS. Afterward, cells were collected, added to stain buffer (BD Biosciences), and incubated with CD3 antibodies (300408, BioLegend) at room temperature for 30 minutes. Cells were then collected in the stain buffer and analyzed for CFSE dilution using flow cytometry. The decrease in CFSE fluorescence intensity was used to quantitatively analyze T cell proliferation. CFSE penetrates the cell membrane and covalently binds to intracellular proteins. As cells divide, CFSE is evenly distributed to daughter cells, leading to a gradual decrease in fluorescence intensity with increasing cell division.

### 2.6 Statistical Analysis

Data have been expressed as mean ± standard deviation. Comparisons between groups were performed using one-way Analysis of Variance (ANOVA), followed by Tukey's post-hoc test. All statistical analyses were conducted using GraphPad Prism 8 software (GraphPad Software, CA, USA). A *p*-value < 0.05 was considered statistically significant.

## 3 RESULTS

### 3.1 Differential IL-8 secretion between Normal Lung Epithelial Cells and Lung Cancer Cells

**Fig. (1):**
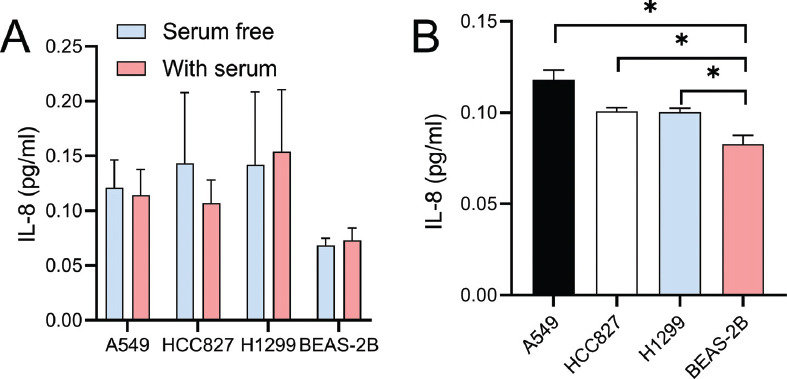
Differential IL-8 secretion in lung cells. (**A**) ELISA analysis of IL-8 secretion under 10% FBS conditions in normal lung epithelial cells and lung cancer cell lines. The graph shows no significant difference in IL-8 secretion with or without FBS, indicating no FBS interference in IL-8 secretion. (**B**) Comparative ELISA analysis of IL-8 secretion between normal lung epithelial cells BEAS-2B and lung cancer cell lines A549, HCC827, and H1299. The graph demonstrates significantly higher IL-8 secretion in lung cancer cell lines, especially A549. *P <0.05.

Through ELISA detection, no significant difference in IL-8 secretion was observed under conditions with or without 10% FBS (*P >* 0.05) (Fig. **1A**), ruling out the interference of FBS in the experiment. Compared to normal lung epithelial cells BEAS-2B, lung cancer cell lines A549, HCC827, and H1299 exhibited significantly higher IL-8 secretion (*P <* 0.05), especially A549; thus, A549 cell-conditioned media was selected for subsequent studies (Fig. **1B**).

### 3.2 Impact of different Interventions on Neutrophil Apoptosis

**Fig. (2):**
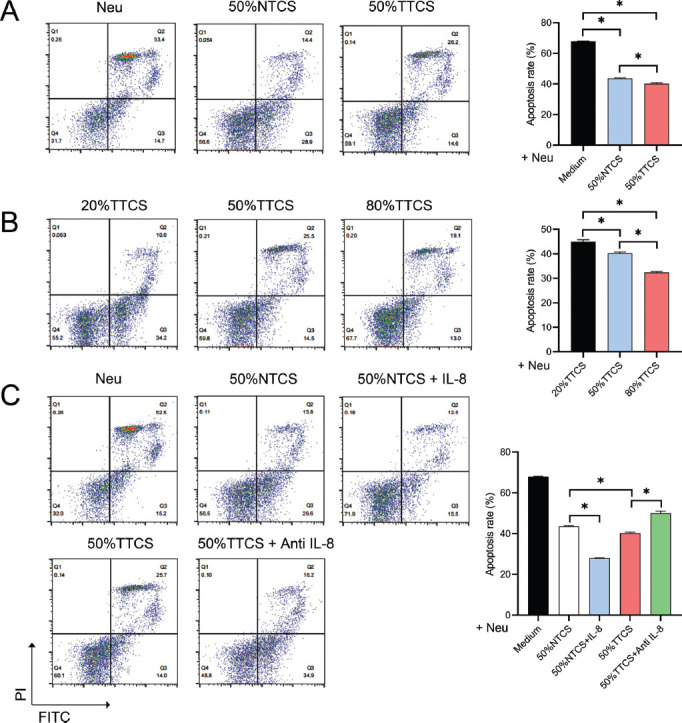
Neutrophil apoptosis under different interventions. (**A**) Flow cytometry analysis of neutrophil apoptosis after treatment with 50% NTCS and TTCS. The graph shows a significant reduction in apoptosis, particularly with TTCS. (**B**) Dose-dependent analysis of neutrophil apoptosis with varying concentrations of TTCS. The graph indicates a significant decrease in apoptosis with increasing TTCS concentration. (**C**) Effect of IL-8 and anti-IL-8 on neutrophil apoptosis in the presence of 50% TTCS. The graph shows a reduction in apoptosis with IL-8 and an increase with anti-IL-8. *P < 0.05.

After isolating neutrophils from normal peripheral blood and culturing them in RPMI-1640 medium, flow cytometry was used to analyze the apoptosis of neutrophils treated with different concentrations of A549 cell-conditioned media and IL-8 inhibitor. Compared to the control group, the apoptosis level of neutrophils significantly decreased after intervention with 50% NTCS and TTCS (*P <* 0.05), particularly with TTCS (Fig. **2A**). Further intervention with varying concentrations of TTCS showed a significant decrease in neutrophil apoptosis rate with increasing TTCS concentration (*P <* 0.05) (Fig. **2B**). The addition of IL-8 led to a significant reduction in apoptosis compared to 50% NTCS (*P <* 0.05), but the addition of anti-IL-8 in 50% TTCS increased the apoptosis level (*P <* 0.05) (Fig. **2C**).

### 3.3 Impact of Different Interventions on PD-L1 Expression in Neutrophils

**Fig. (3):**
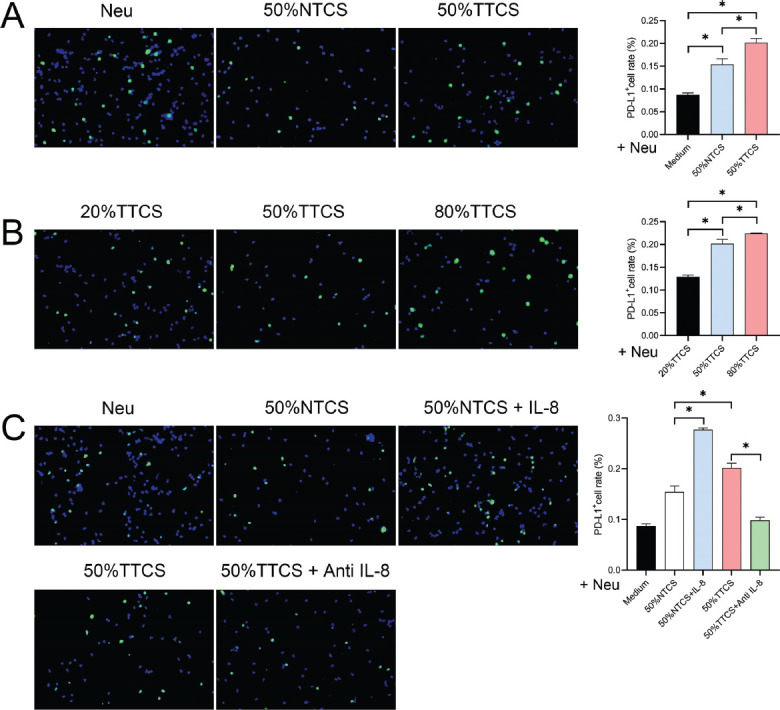
PD-L1 expression in neutrophils under different interventions. (**A**) Immunofluorescence analysis of PD-L1 expression in neutrophils treated with 50% NTCS and TTCS. The graph shows a significant increase in PD-L1 expression, especially with TTCS. (**B**) Dose-dependent analysis of PD-L1 expression in neutrophils with varying concentrations of TTCS. The graph indicates a gradual increase in PD-L1 expression with increasing TTCS concentration. (**C**) Effect of IL-8 and anti-IL-8 on PD-L1 expression in the presence of 50% TTCS. The graph shows an increase in PD-L1 expression with IL-8 and a decrease with anti-IL-8. *P < 0.05.

Immunofluorescence was used to analyze PD-L1 expression in neutrophils treated with different concentrations of cell-conditioned media and IL-8 inhibitor. Compared to the control group, PD-L1 expression in neutrophils significantly increased after intervention with 50% NTCS and TTCS (*P <* 0.05), especially with TTCS (Fig. **3A**). Further intervention with varying concentrations of TTCS showed a gradual significant increase in PD-L1 expression in neutrophils with increasing TTCS concentration (*P <* 0.05) (Fig. **3B**). The addition of IL-8 led to a significant increase in PD-L1 expression compared to 50% NTCS (*P <* 0.05), but the addition of anti-IL-8 in 50% TTCS significantly reduced PD-L1 expression (*P <* 0.05) (Fig. **3C**).

### 3.4 Impact of Different Interventions on PD-L1+ Neutrophils

**Fig. (4):**
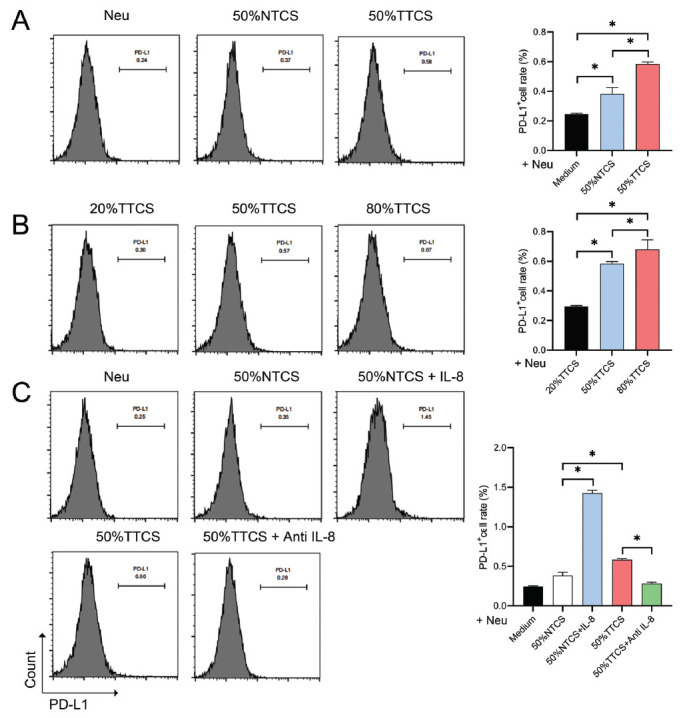
Impact of different interventions on PD-L1+ neutrophils. (**A**) Flow cytometry analysis of PD-L1 expression after treatment with 50% NTCS and TTCS. The graph shows a significant increase in PD-L1 expression, particularly with TTCS. (**B**) Dose-dependent analysis of PD-L1+ neutrophils with varying concentrations of TTCS. The graph indicates a significant increase in PD-L1 expression with increasing TTCS concentration. (**C**) Effect of IL-8 and anti-IL-8 on PD-L1+ neutrophils in the presence of 50% TTCS. The graph shows an increase in PD-L1 expression with IL-8 and an increase with anti-IL-8. *P < 0.05.

Flow cytometry was used to analyze PD-L1 expression in neutrophils treated with different concentrations of cell-conditioned media and IL-8 inhibitor. Compared to the control group, PD-L1 expression in neutrophils significantly increased after intervention with 50% NTCS and TTCS (*P <* 0.05), especially with TTCS (Fig. **4A**). Further intervention with varying concentrations of TTCS showed a gradual significant increase in PD-L1 expression in neutrophils with increasing TTCS concentration (*P <* 0.05) (Fig. **4B**). The addition of IL-8 led to a significant increase in PD-L1 expression compared to 50% NTCS (*P <* 0.05), but the addition of anti-IL-8 in 50% TTCS significantly reduced PD-L1 expression (*P <* 0.05) (Fig. **4C**).

### 3.5 Impact of Neutrophils on CD3+ T Cell Proliferation

**Fig. (5):**
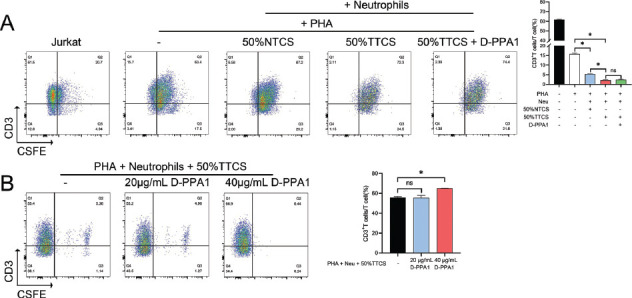
Effect of neutrophils on CD3+ T cell proliferation. CFSE staining analysis of CD3+ T cell proliferation under the influence of neutrophils and various interventions. (**A**) The graph shows a reduction in T cell proliferation with the addition of neutrophils, particularly with 50% TTCS. (**B**) An increase in proliferation with the addition of D-PPA1 (40 μg/ml). *P < 0.05; ns, no significance.

CFSE results indicated that compared to Jurkat cells, the addition of PHA reduced CD3+ T cell proliferation. Further addition of neutrophils with other intervention measures reduced CD3+ T cell proliferation levels (*P <* 0.05), with the addition of 50% TTCS and 50% TTCS+D-PPA1 being the most significant (Fig. **5A**). Additionally, compared to the Jurkat + PHA + neutrophils + 50% A549 group, the further addition of 40 μg/mL D-PPA1 significantly increased CD3+ T cell proliferation (*P <* 0.05), but no significant difference was observed with the addition of 20 μg/mL D-PPA1 (*P >*0.05) (Fig. **5B**).

### 3.6 Impact of Neutrophils on IFN-γ Levels

**Fig. (6):**
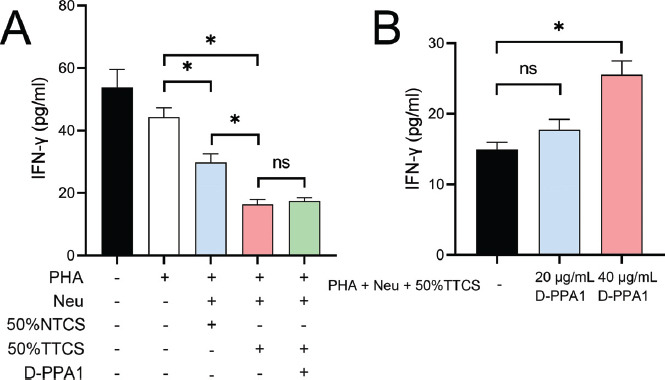
Influence of neutrophils on IFN-γ levels. ELISA analysis of IFN-γ secretion in T cells after treatment with neutrophils and various interventions. (**A**) The graph shows a reduction in IFN-γ secretion with the addition of PHA and further reduction with the addition of 50% TTCS. (**B**) The addition of D-PPA1 (40 μg/ml) led to an increase in IFN-γ levels. *P < 0.05; ns, no significance.

ELISA results showed that compared to Jurkat cells, the addition of PHA reduced IFN-γ secretion. Further addition of neutrophils with other intervention measures reduced IFN-γ levels (*P <* 0.05), with the addition of 50% TTCS and 50% TTCS+D-PPA1 being the most significant (Fig. **6A**). Additionally, compared to the Jurkat + PHA + neutrophils + 50% TTCS group, the further addition of 40 μg/mL D-PPA1 significantly increased IFN-γ levels (*P <* 0.05), but no significant difference was observed with the addition of 20 μg/mL D-PPA1 (*P >*0.05) (Fig. **6B**).

## 4 DISCUSSION

In this study, we have first eliminated the potential interference of FBS in our experimental outcomes. More importantly, lung cancer cell lines, particularly A549, exhibited higher IL-8 secretion compared to normal lung epithelial cells BEAS-2B. This observation aligns with previous studies suggesting that lung cancer cells may promote an inflammatory environment through enhanced IL-8 secretion [49], closely related to lung cancer's inflammatory characteristics and immune regulatory functions [50]. In alignment with the multidimensional analysis conducted by Liu *et al*. [51], which identified consistently differentially expressed hub mRNAs and proteins in lung adenocarcinoma, our study has further emphasized the importance of understanding the tumor microenvironment's role in modulating immune responses. The differential expression of key proteins, such as those involved in immune escape mechanisms, underscores the complexity of lung cancer progression and the potential for targeted therapeutic strategies. As a typical pro-inflammatory cytokine, IL-8 not only plays a role in regulating local inflammatory responses, but may also participate in tumor growth, invasion, and metastasis by affecting immune cells, such as TANs, in the tumor microenvironment [52-54]. Subsequently, we have observed that interventions with 50% cell-conditioned media, particularly TTCS, dose-dependently reduced neutrophil apoptosis, indicating that certain components in TTCS might inhibit neutrophil apoptotic pathways. As part of the immune system, reduced apoptosis in neutrophils might lead to their accumulation in the tumor microenvironment, thereby promoting tumor growth and metastasis [29, 55-57]. Unlike previous studies, we are the first to note that IL-8 further inhibited the cytotoxicity of NTCS on neutrophils, while its inhibitor partially counteracted the apoptosis inhibition effect of TTCS on neutrophils, further confirming the role of IL-8 in the regulation of neutrophil apoptosis. The inclusion of NTCS in our experimental design was crucial for delineating the specific effects of tumor-derived factors on neutrophil functions. Our findings have indicated NTCS to provide a comparative baseline, as in a previous study [58], which demonstrated the observed changes in neutrophil behavior to be more pronounced with TTCS. This highlights the unique role of the tumor microenvironment in modulating immune responses, further underscoring the significance of NTCS in the study.

Our results have shown the PD-L1 levels in neutrophils to be significantly increased under the influence of TTCS, consistent with previous studies [58], reflecting lung cancer cells' ability to modulate the immune microenvironment and enhance immune escape capabilities. PD-L1, by binding to PD-1 on T cells, can help tumor cells evade the immune system's attack [43, 59-63]. The difference is that IL-8 has been observed to further enhance the PD-L1 expression by NTCS, while its inhibitor has partially negated the promoting effect of TTCS on PD-L1 expression. This result suggests that IL-8 might be one of the key factors regulating PD-L1 expression in neutrophils, and its inhibition could disrupt the interaction between lung cancer cells and neutrophils, thereby reducing the immunosuppressive function of neutrophils. The flow cytometry results have been found to be consistent with our immunofluorescence data, further confirming that lung cancer cell-conditioned media can significantly increase PD-L1 expression in neutrophils. It is noteworthy that both NTCS and TTCS groups showed significant differences compared to the control group across several parameters, including IL-8 and IFN-γ secretion, apoptosis, PD-L1 expression, and T cell proliferation. While the differences between the NTCS and TTCS groups were meaningful, they were less pronounced than those observed between the control group and the experimental groups. This observation suggests that components present in the culture medium itself may influence neutrophil behavior to some extent. These effects, however, appear to be more pronounced when the culture medium is derived from tumor tissue, as seen in the TTCS group. Therefore, while the culture medium can modulate neutrophil functions, the tumor-specific factors in TTCS likely play a more significant role in driving the observed changes.

PHA is a lectin that has been shown in previous studies to bind to T-cell membranes, thereby increasing metabolic activity and cell division. It is widely used by researchers to activate T cells [64-67]. Under PHA conditions, we found that the addition of neutrophils further reduced the proportion of CD3+ T cells, especially when 50% TTCS was added. This result indicates that factors in lung cancer cell-conditioned media can not only directly affect neutrophil functions, but also indirectly influence T cell activity, further revealing the complex immune regulatory network within the lung cancer microenvironment. When the PD-L1 inhibitor D-PPA1 was additionally introduced, the proportion of CD3+ T cells increased, confirming the inhibition of Jurkat by PD-L1. This finding provides a potential strategy to enhance T cell immune responses against lung cancer, as the suppression of T cell proportion leads to immune escape of lung cancer cells [68, 69], which is likely a mechanism for evading immune surveillance.

Jurkat cells are often used as a model system to study T-cell activation and cytokine production and for drug screening studies because they express the CD3 complex on their surface, which is part of the T-cell Receptor (TCR) complex and is essential for T-cell activation and signaling [70-73]. We observed that PHA reduced IFN-γ secretion, and further addition of neutrophil intervention, especially with 50% TTCS, further reduced IFN-γ levels. This result suggests that factors in lung cancer cell-conditioned media not only affect neutrophil functions, but also indirectly influence T cell IFN-γ secretion, potentially affecting T cell-mediated immune responses [74-77]. IFN-γ, as a key immune regulatory cytokine, plays a crucial role in anti-tumor immunity [78], and thus, lung cancer cells' modulation of neutrophils to reduce IFN-γ secretion might be part of their immune evasion strategy. Further experiments have also found that when D-PPA1 was additionally introduced, IFN-γ levels increased, indicating that inhibition of PD-L1 can partially reverse the suppressive effect of lung cancer cell-conditioned media on IFN-γ secretion. Therefore, neutrophils, influenced by factors in lung cancer cell-conditioned media, might play a significant role in immune evasion in lung cancer through their impact on T cell IFN-γ secretion.

Our study has involved some limitations. First, it primarily relied on *in vitro* experimental models, not fully replicating the complex tumor microenvironment and immune system dynamics *in vivo*. Second, while our study has mainly focused on the role of IL-8 in regulating neutrophil functions, lung cancer cell-conditioned media contain a variety of factors that may collectively act on neutrophils. Third, our study has primarily focused on neutrophil apoptosis, PD-L1 expression, and their impact on T cell functions, but neutrophil functions extend beyond these aspects. Neutrophils can also participate in tumor development and immune regulation through other mechanisms, such as forming NETs and producing reactive oxygen species. Fourth, the exclusion of a control group with only PHA and Neu may limit the ability to discern the independent effects of 50% NTCS and 50% TTCS. Finally, the lung cancer microenvironment includes a variety of other immune cells, such as T cells, B cells, and macrophages. The interactions among these cells and their interactions with lung cancer cells constitute a complex immune network. Future research needs to overcome these limitations by conducting *in vivo* experiments, exploring the interactions of various factors, comprehensively assessing the multiple functions of neutrophils, and considering the interactions of various immune cells in the lung cancer microenvironment to provide more information and strategies for lung cancer treatment.

## CONCLUSION

In conclusion, our study has systematically explored the impact of lung cancer cell-conditioned media on neutrophil functions and their potential role in the lung cancer immune microenvironment. Our findings have revealed as to how lung cancer cells modulate neutrophil apoptosis, PD-L1 expression, and impact on T cell functions through regulating factors, like IL-8, providing new perspectives for understanding the immune regulatory mechanisms within the lung cancer microenvironment and potentially offering clues for developing new lung cancer treatment strategies.

## AUTHORS’ CONTRIBUTIONS

YZ and JC designed the experiments. QJ, LL, and KL performed the experiments. LD and JG helped to collect the data. JC and YH analyzed and interpreted the data. YZ and YH drafted the manuscript. All authors have read and approved the final manuscript.

## Data Availability

The datasets used or analysed during the current study will be available from the corresponding author upon reasonable request.

## LIST OF ABBREVIATIONS

BSA: Bovine Serum Albumin
CFSE: Carboxyfluorescein Diacetate Succinimidyl Ester
FBS: Fetal Bovine Serum
IFN-γ: Interferon Gamma
IL-8: Interleukin 8
MHC: Major Histocompatibility Complex
NETs: Neutrophil Extracellular Traps
NTCS: Non-tumor Tissue Culture Supernatant
PBS: Phosphate Buffered Saline
PD-L1: Programmed Death-ligand 1
PHA: Phytohemagglutinin
PI: Propidium Iodide
RPMI-1640: Roswell Park Memorial Institute 1640 Medium
TCR: T-cell Receptor
TTCS: Tumor Tissue Culture Supernatant
